# Evaluation of contrast wash-in and peak enhancement in adenosine first pass perfusion in patients post bypass surgery

**DOI:** 10.1186/1532-429X-11-S1-O39

**Published:** 2009-01-28

**Authors:** Christoph Klein, Eike Nagel, Kristof Graf, Stephan Dreysse, Bernhard Schnackenburg, Eckart Fleck

**Affiliations:** 1 German Heart Institute Berlin, Berlin, Germany; 2 King's College London, London, UK; 3 Philips Medical Systems, Hamburg, Germany

**Keywords:** Coronary Artery Bypass Graft, Late Gadolinium Enhancement, Invasive Coronary Angiography, Significant Coronary Artery Disease, Peak Enhancement

## Introduction

CMR adenosine first pass perfusion yields excellent results for the detection of significant coronary artery disease. As in patients after coronary artery bypass grafts (CABG) myocardial perfusion is more complex and additionally the kinetic of a first pass bolus may by altered due to different distances through the bypasses and/or native vessels, this patient group has been excluded from most published studies. Parameters like speed of contrast wash-in (upslope, time to 50% or peak enhancement) and peak enhancement are indirectly used for visual analysis and may be altered post CABG imitating perfusion defects without significant stenosis. In this case, adenosine perfusion would be an inadequate diagnostic test.

## Purpose

Aim of the study was to evaluate semiquantitative perfusion parameters in patients after CABG in order to evaluate contrast kinetics in areas supplied by native coronaries and different bypass grafts.

## Methods

32 patients post CABG were included into the study consisting of adenosine first pass (0.05 mmol/kg Gd-DTPA) perfusion (3 short axis views/heart beat) and late Gadolinium enhancement before undergoing invasive coronary angiography. In invasive angiography, areas perfused by native coronaries and the different bypasses were identified. In these areas upslope, time to 50% peak enhancement, time to peak enhancement and relative peak enhancement were calculated using the ViewForum (Philips Medical Systems, Best, Netherlands). Only segments without vessel stenosis and without LGE were used for final analysis.

## Results

**Figure 1:**
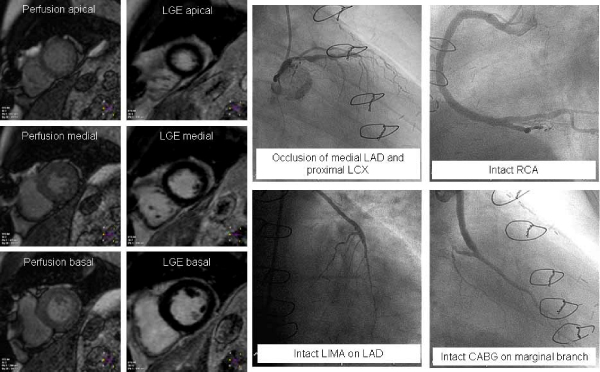
Figure 1

Results are displayed in Table 1. No significant differences in any parameter comparing native vessels with CABG or CABG with CABG were found. Figure 1 shows homogenous perfusion enhancement in a patient post CABG with areas perfused by native RCA, LIMA on LAD and venous graft on a marginal branch.

**Table 1:** Semiquantitative parameters in native vessels and grafts

	Native vessel	CABG	p (native vs. CABG)	Correlation (native vs. CABG)	p (CABG vs. CABG)	Correlation (CABG vs. CABG)
Upslope	17.7 ± 7.8	17.3 ± 7.0	>0.05	0.92	>0.05	0.89
Time to 50%	8.2 ± 1.8	8.1 ± 1.8	>0.05	0.94	>0.05	0.90
Time to peak	13.7 ± 3.1	13.7 ± 3.1	>0.05	0.97	>0.05	0.92
Relative peak enhancement	22.9 ± 9.2	22.0 ± 8.7	>0.05	0.91	>0.05	0.86

## Conclusion

Semiquantitaive parameters of first pass adenosine perfusion are similar in areas supplied by native vessels or by different bypass grafts. These parameters are indirectly used for visual analysis (speed of contrast wash-in and peak signal intensity). Therefore the possible different contrast kinetic through grafts and native vessel does not seem to be a limiting factor for the accuracy of first pass adenosine perfusion in patients post CABG.

